# Comparison of Gut Microbiota and Metabolic Status of Sows With Different Litter Sizes During Pregnancy

**DOI:** 10.3389/fvets.2021.793174

**Published:** 2021-12-23

**Authors:** Jiali Chen, Fuchang Li, Weiren Yang, Shuzhen Jiang, Yang Li

**Affiliations:** Shandong Provincial Key Laboratory of Animal Biotechnology and Disease Control and Prevention, Department of Animal Science and Veterinary Medicine, Shandong Agricultural University, Tai'an, China

**Keywords:** gestation stage, gut microbiota, litter size, metabolic status, reproductive performance, sow

## Abstract

The experiment was conducted to compare the differences of gut microbiota and metabolic status of sows with different litter sizes on days 30 and 110 of gestation, and uncover the relationship between the composition of maternal gut microbiota during gestation and sow reproductive performance. Twenty-six Large White × Landrace crossbred multiparous sows (2nd parity) with similar back fat thickness and body weight were assigned to two groups [high-reproductive performance group (HP group) and low-reproductive performance group (LP group)] according to their litter sizes and fed a common gestation diet. Results showed that compared with LP sows, HP sows had significantly lower plasma levels of triglyceride (TG) on gestation d 30 (*P* < 0.05), but had significantly higher plasma levels of TG, non-esterified fatty acid, tumor necrosis factor-α, and immunoglobulin M on gestation d 110 (*P* < 0.05). Consistently, HP sows revealed increased alpha diversity and butyrate-producing genera, as well as fecal butyrate concentration, on gestation d 30; HP sows showed significantly different microbiota community structure with LP sows (*P* < 0.05) and had markedly higher abundance of Firmicutes (genera *Christensenellaceae_R-7_group* and *Terrisporobacter*) which were positively related with litter size on gestation d 110 than LP sows (*P* < 0.05). In addition, plasma biochemical parameters, plasma cytokines, and fecal microbiota shifted dramatically from gestation d 30 to d 110. Therefore, our findings demonstrated that microbial abundances and community structures differed significantly between sows with different litter sizes and gestation stages, which was associated with changes in plasma biochemical parameters, inflammatory factors, and immunoglobulin. Moreover, these findings revealed that there was a significant correlation between litter size and gut microbiota of sows, and provided a microbial perspective to improve sow reproductive performance in pig production.

## Introduction

Diverse microbial communities reside at various sites within a mammalian body (1, 2). Gut microbiota makes up the vast majority of body's microbes and with an estimated number of several trillion most probably outnumber human body cells (3). The gut microbiota is shaped by many environmental factors, such as host genetics (4), diet (5), and the immune system (6), and has been reported to play a vital role in inflammation, metabolic syndrome (7), energy metabolism (8), and immunity (9).

Previous study in humans showed that the body experiences extensive hormonal, metabolic, and immunological changes over the course of normal and healthy pregnancy (10), accompanied by dramatic changes in maternal gut microbiota (11). Koren et al. (10) showed normal pregnancy to be accompanied by a profound change of gut microbiota from the first to the third trimester with an increase in the Proteobacteria and Actinobacteria abundances which might be connected with the maternal metabolic profile. Uryu et al. (12) demonstrated that sow productivity on different farms was likely related to changes in fecal microbe composition. Besides, research showed that dietary probiotic supplementation in gestating sow diet could increase the number of piglets total born (13, 14). Further, Al-Asmakh et al. (15) found that maternal microbiota could regulate placental development and then might affect the development of the growing offspring in mice. This research suggests that maternal gut microbiota during gestation is affecting sow reproductive performance. However, there is little literature available about whether the composition of gut microbiota during gestation is associated with improved sow reproductive performance.

The early and late pregnancy are two critical stages for embryonic survival and development (16, 17). In the present study, we aimed to explore the relationship between reproductive performance and maternal gut microbiota during gestation through comparing the fecal microbiota characteristics and metabolic status of sows with high (>12 piglets per litter) and low litter size (≤ 12 piglets per litter) on day 30 of gestation (G30) and on day 110 of gestation (G110).

## Materials and Methods

### Ethical Approval

This study was conducted at the pig breeding farm in Shandong Province. The animal use protocol for this research was approved by the Animal Care and Use Committee of Shandong Agricultural University (Approval Number: SDAUA-2019-019).

### Animals and Experimental Design

Twenty-six Large White × Landrace crossbred multiparous sows (2nd parity) with similar back fat thickness (BF, 15.28 ± 0.45 mm) and body weight (174.34 ± 2.72 kg) were used in this study. The BF at the last rib was measured using a HG 9300 digital diagnostic ultrasound device (Caresono Technology Co. Ltd., Nanjing, China). After artificial insemination, the individual sow was housed individually in a gestation stall (2.37 × 0.65 × 1.13 m) kept at 21 ± 1°C. All the sows were mated within 3 days and fed a common fortified corn–soybean meal gestation diet (Supplementary Table 1) which was formulated to meet or exceed National Research Council (18) nutrient requirements. All sows received a daily meal at 0900 h and were fed the same amount of feed (days 1 to 89 of gestation 2.46 kg/d; days 90 of gestation to farrowing, 2.89 kg/d) during the entire gestation. On day 110 of gestation, sows were moved from gestation to farrowing rooms and kept in individual farrowing crates measuring 2.40 × 1.80 × 0.90 m thereafter. Backfat thickness and body weight of individual sow were measured at breeding and within 24 h of farrowing. At farrowing, the numbers of total born piglets, live born piglets, and dead born piglets per litter, as well as litter weight, were recorded, and the averages were calculated. Thus, two groups were generated (Table 1): 13 sows with litter size lower than the average in this trial (12.7 piglets) were classified as the low-reproductive performance group (LP group), while 13 sows with litter size higher than the average in this trial (12.7 piglets) labeled as the high-reproductive performance group (HP group). Sows had free access to water throughout the experiment and did not receive vaccine, antibiotics, or other medication in the feed or for any therapeutic purposes after insemination.

**Table 1:** Litter size and litter weight in low- and high-reproductive performance groups.

**Items**	**Group^a^**		***P*** **-value**
	**LP**	**HP**	
No. of sows	13	13	-
Backfat thickness, mm			
Breeding	15.33 ± 0.74	14.84 ± 0.53	0.599
Farrowing	19.16 ± 0.72	17.82 ± 0.77	0.218
**Body weight, kg**			
Breeding	179.32 ± 4.34	175.31 ± 4.18	0.631
Farrowing	238.77 ± 3.50	231.84 ± 3.91	0.199
**Litter size**			
Total born	9.77 ± 0.53	15.54 ± 0.66	<0.001
Born alive	9.46 ± 0.57	14.31 ± 0.61	<0.001
Dead	0.31 ± 0.13	1.23 ± 0.30	0.013
**Litter weight, kg**			
Total born	16.55 ± 0.64	22.16 ± 0.84	<0.001
Born alive	16.03 ± 0.66	21.21 ± 0.83	<0.001

a *LP, Sows in low-Reproductive Performance Group; HP, Sows in High-Reproductive Performance Group.*

*Values are mean ± standard error (n = 13)*.

### Sample Collection

Fasting blood samples (12 h overnight) and fresh fecal samples from all healthy sows were collected on day 30 and day 110 of gestation before feeding in the morning. Samples were grouped as follows: LP30 and LP110: sows with low-reproductive performance on day 30 and day 110 of gestation, respectively; HP30 and HP110: sows with high-reproductive performance on day 30 and day 110 of gestation, respectively. Blood samples (5 mL) from the ear veins were collected into a tube containing heparin sodium and centrifuged at 3,000× g for 15 min. Plasma samples was transferred to 200 μL centrifuge tubes and stored at −20°C until analysis. Fecal samples (about 5 g) were collected from the rectum by a sterilized fecal collection tube and then stored at −80°C immediately for the detection of short-chain fatty acids (SCFAs) and analysis of microbiota.

### Plasma Biochemical Parameters Analysis

Plasma biochemical parameters, including glucose (GLU), cholesterol (CHOL), triglyceride (TG), high density lipoprotein cholesterol (HDL-C), low density lipoprotein cholesterol (LDL-C), and non-esterified fatty acid (NEFA), were determined with commercial kits (Nanjing Jiancheng Bioengineering Institute, Nanjing, China) using standard spectrophotometric methods on an Autolab-PM4000 Automatic Analyzer (AMS Co., Rome, Italy) as previously described (19).

### Analysis of Inflammatory Factors, Immunoglobulins, and Reproductive Hormones

Concentrations of interleukin-2 (IL-2), interleukin-6 (IL-6), interleukin-10 (IL-10), tumor necrosis factor-α (TNF-α), immunoglobulin A (IgA), immunoglobulin G (IgG), immunoglobulin M (IgM), progesterone, estrogen, lutropin, and prolactin in the plasma of sows were determined with commercial ELISA kits (Feiya Biotechnology Co. Ltd., Yancheng, China) as described in Supplementary Methods.

### Determination of Fecal SCFAs

The fecal SCFAs of sows were measured by a Varian CP-3800 gas chromatograph (Palo Alto, CA, USA) equipped with a micro-injector, a flame ionization detector, and a capillary chromatographic column as described in Supplementary Methods.

### Microbial Analysis

Microbial composition and diversity were analyzed as previously described in Li et al. (20). Briefly, bacterial genomic DNA was extracted from frozen fecal samples with an E.Z.N.A.^TM^ Stool DNA kit (Omega Bio-Tek, Norcross, GA, USA) according to the manufacturer's protocol. After DNA concentration and purity monitoring, DNA was diluted to 1 ng/μL using sterile water, and the V4 hypervariable region of 16S rDNA was amplified with 515F and 806R primer (5′-GTGCCAGCMGCCGCGGTAA-3′ and 5′-GGACTACHVGGGTWTCTAAT-3′, respectively), on the Illumina HiSeq PE2500 platform by Novogene (Beijing, China). Filtered, non-chimeric high-quality sequences (tags) sharing over 97% sequence similarity were clustered into the same operational taxonomic units (OTUs) by Uparse software (21), and then classified to different taxonomic levels with SILVA database (22) based on Mothur algorithm to annotate taxonomic information. Operational taxonomic units abundance information were normalized using a standard of sequence number corresponding to the sample with the least sequences for subsequent analysis of alpha diversity and beta diversity. Shannon, Simpson, Chao 1, and ACE indexes were chosen to ascertain differences in alpha diversity based on different groups (23, 24), and Bray-Curtis distances were calculated and visualized using Principal Coordinate Analysis (PCoA) (25). The statistical differences in alpha and beta diversity of bacterial communities between the two groups were examined using the Wilcoxon rank-sum test. Significant difference among the microbial communities was accessed with the analysis of similarity (ANOSIM) test.

### Statistical Analysis

The individual sow was regarded as the experimental unit for all variables. Differences in the data including plasma biochemical parameters, inflammatory factors, and fecal SCFAs were evaluated using the independent *t*-test (LP vs. HP) or paired *t*-test (G30 vs. G110) procedure of SAS 9.0 (Institute Inc., Cary, NC, USA) following normal distribution assessment using a Shapiro-Wilk's statistic (*W* > 0.05). Multiple testing was corrected by using the Benjamini-Hochberg false discovery rate. Spearman's correlations were used to assess the associations between bacterial abundance and litter size, as well as plasma biochemical indices. Treatment differences were considered statistically significant at *P* < 0.05, and 0.05 ≤ *P* < 0.10 was considered a statistical trend. Values are expressed as mean ± standard error in tables and figures.

## Results

### Changes of Fecal Microbial Diversity

**Figure 1:**
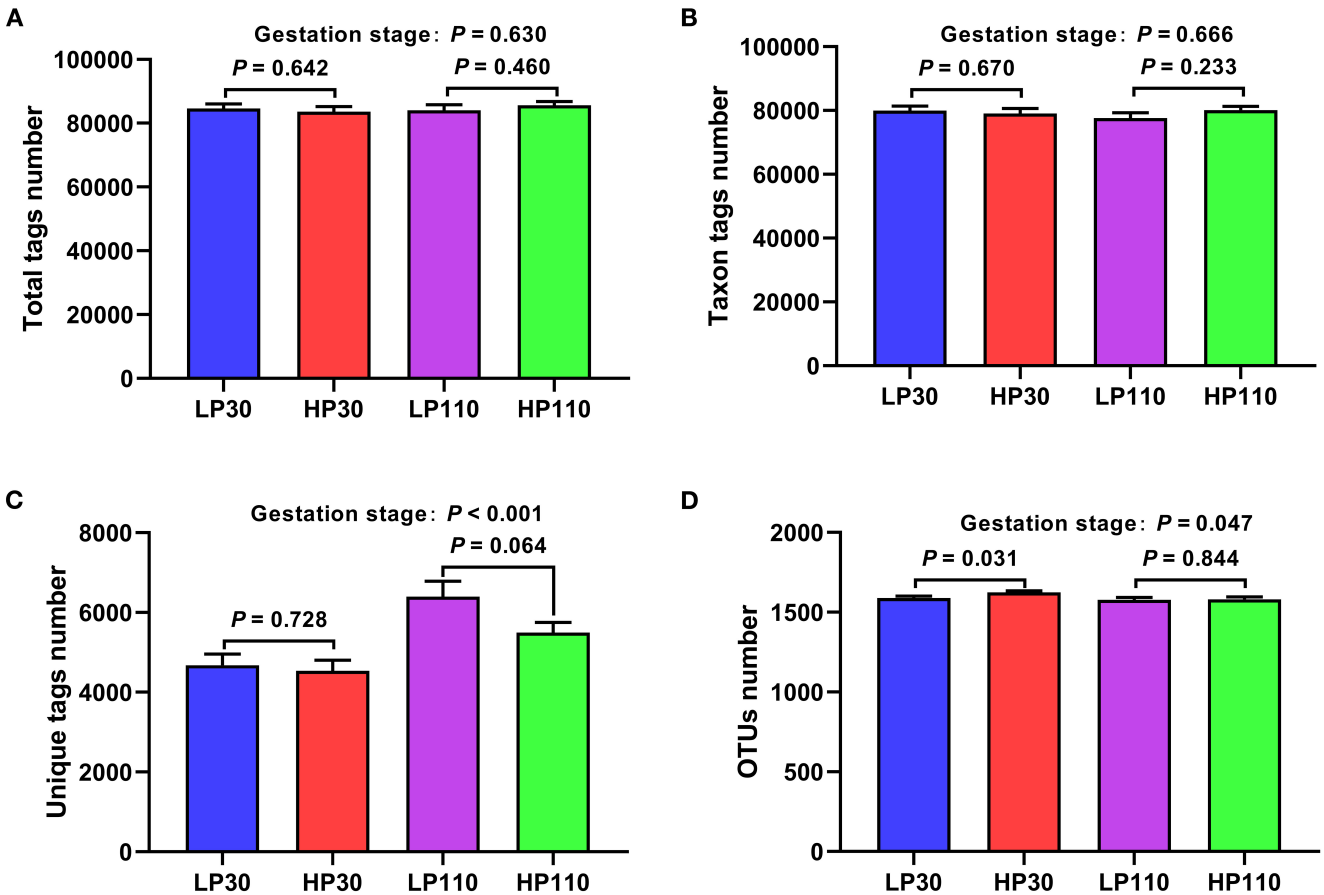
Operational taxonomic unit (OUT) clustering and annotation of sow fecal samples on d 30 and d 110 of gestation. **(A)** Total tags number; **(B)** taxon tags number; **(C)** unique tags number; **(D)** OTUs number. LP30 and LP110: sows with low-reproductive performance on d 30 and d 110 of gestation, respectively; HP30 and HP110: sows with high-reproductive performance on d 30 and d 110 of gestation, respectively. Gestation stage: difference in the variations between gestation d 30 and d 110. Values are mean ± standard error (*n* = 13).

A total of 4,392,562 total tags, 4,118,486 taxon tags, and 274,447 unique tags were obtained from 52 sow fecal samples, with an average of 84,472 ± 730, 79,202 ± 720 and 5,278 ± 179 per sample, respectively (Figures 1A–C). Based on 97% sequence similarity, a total of 21,114 OTUs were found in the HP group on day 30 of gestation, with an significantly higher average of 1,624 ± 10 OUT per sample compared to an average of 1,589 ± 12 OUT per sample in the LP group, where 20,657 OTUs were found in total (Figure 1D); the HP group tended had lower unique tags than the LP group on day 110 of gestation (5,500 ± 248 vs. 6,397 ± 389; *P* = 0.064; Figure 1C). In addition, from gestation d 30 to d 110, the unique tags number was significantly increased on average (*P* < 0.05), but OTUs number was decreased (*P* = 0.047).

To determine whether the sample size was sufficient for OUT testing, the species accumulation curves (SAC) was used in the present study. The SAC (Supplementary Figure 1) tended to flatten as the sample number of analyzed sequences increased up to 52, suggesting that the sample size was enough for OTUs testing and could estimate the species richness of the habitat.

**Figure 2:**
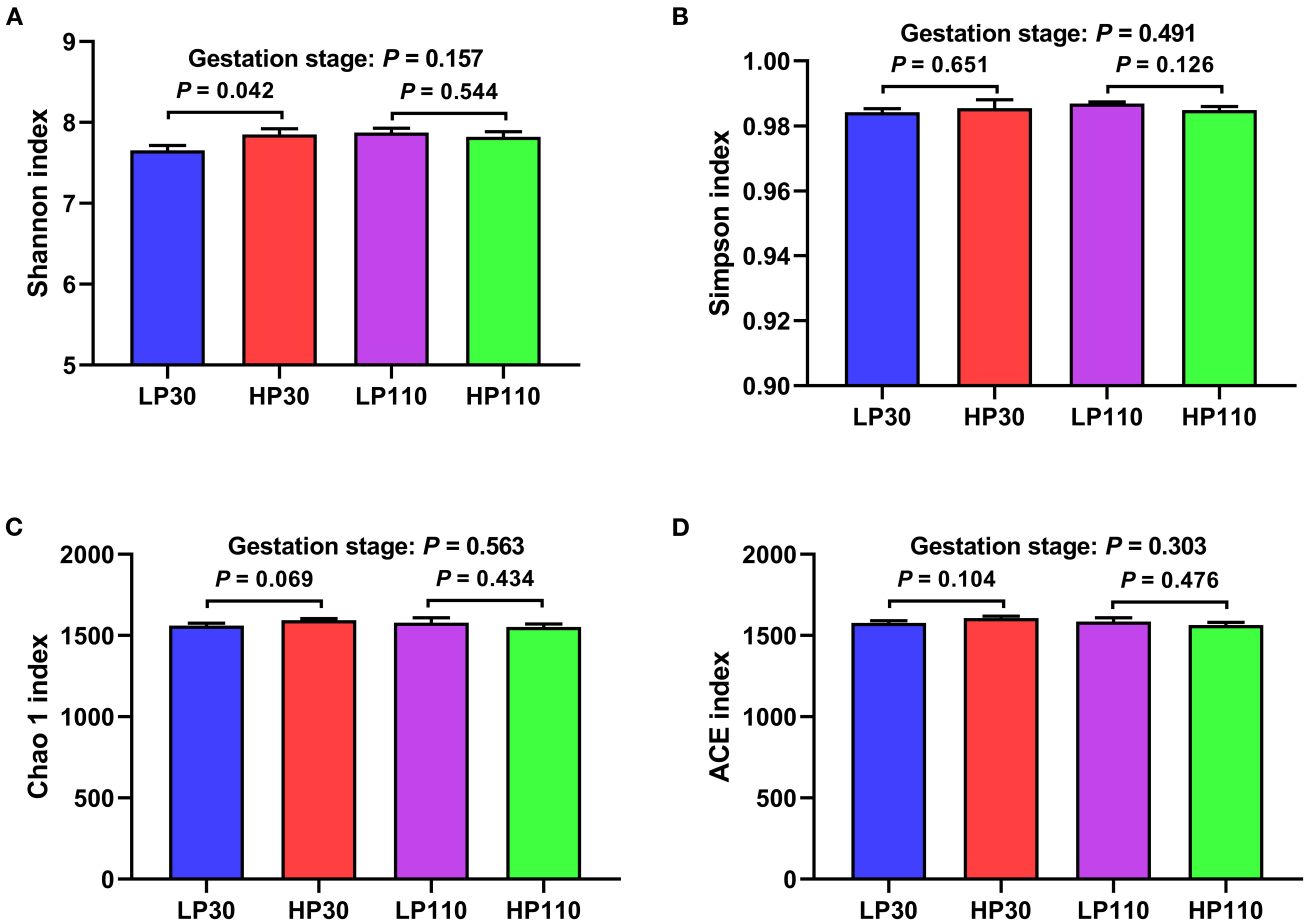
Difference on bacteria community diversity and richness among different groups on d 30 and 110 of gestation. **(A)** Shannon index; **(B)** Simpson index; **(C)** Chao 1 index; **(D)** ACE index. LP30 and LP110: sows with low-reproductive performance on d 30 and 110 of gestation, respectively; HP30 and HP110: sows with high-reproductive performance on d 30 and 110 of gestation, respectively. Gestation stage: difference in the variations between gestation d 30 and 110. Values are mean ± standard error (*n* = 13).

The results of fecal microbial community structures assessment are shown in Figure 2. On d 30 of gestation, the HP group had significantly higher Shannon index (*P* < 0.05) and tended to have a higher Chao 1 index compared with LP group (*P* = 0.069); on d 110 of gestation, no significant differences were observed in alpha diversity between the two groups (*P* > 0.05).

**Figure 3:**
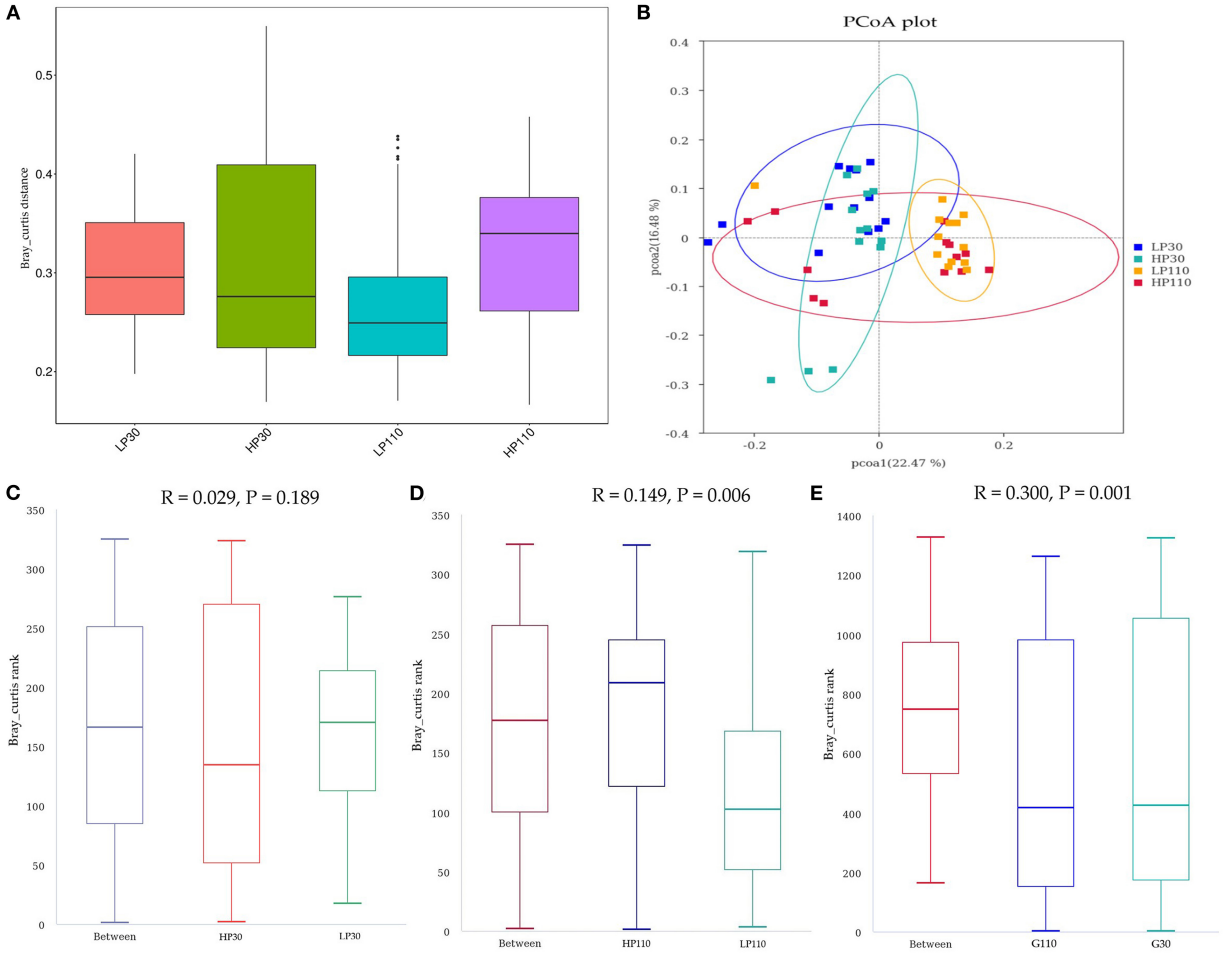
The beta diversity of microbial communities in the groups on d 30 and d 110 of gestation. **(A)** The Bray-Curtis distance within each group. **(B)** The principal coordinate analysis (PCoA) profile of the two groups displayed with the Bray-Curtis distance. Each dot represents one sample from each group. The percent variation explained by each principal coordinate is indicated on the X and Y axis. **(C–E)** Analysis of similarity (ANOSIM). R value is scaled to lie between −1 and +l. Generally, 0 < R < 1 and *P* < 0.05 represents that there were significant differences between the groups. LP30 and LP110: sows with low-reproductive performance on d 30 and d 110 of gestation, respectively; HP30 and HP110: sows with high-reproductive performance on d 30 and d 110 of gestation, respectively. *n* = 13 for each group.

In addition, to measure the evolutionary distance between microbiotas (beta diversity), the PCoA profile for sow fecal samples based on the Bray-Curtis distance was used in the present study, and ANOSIM test was used to assess significant differences among the microbial communities (Figures 3A,B). The results suggested that LP and HP groups had close distance on gestation d 30 which showed that the two groups had no significant difference in the microbial community (*P* = 0.189, Figure 3C); an obvious separation was observed in PCoA between samples from HP group and LP group on d 110 of gestation. The ANOSIM test also indicated that the two groups had notably different microbiota structures on gestation d 110 (*P* = 0.006, Figure 3D), and sows in HP group had greater beta diversity compared to sows in LP group at 110 days of gestation (*P* < 0.05). Besides, the Bray-Curtis distance analysis showed a global shift in microbial community composition from gestation d 30 to d 110 (*P* = 0.001, Figures 3B,E).

### Changes in Relative Abundance of Phyla and Genera

**Figure 4:**
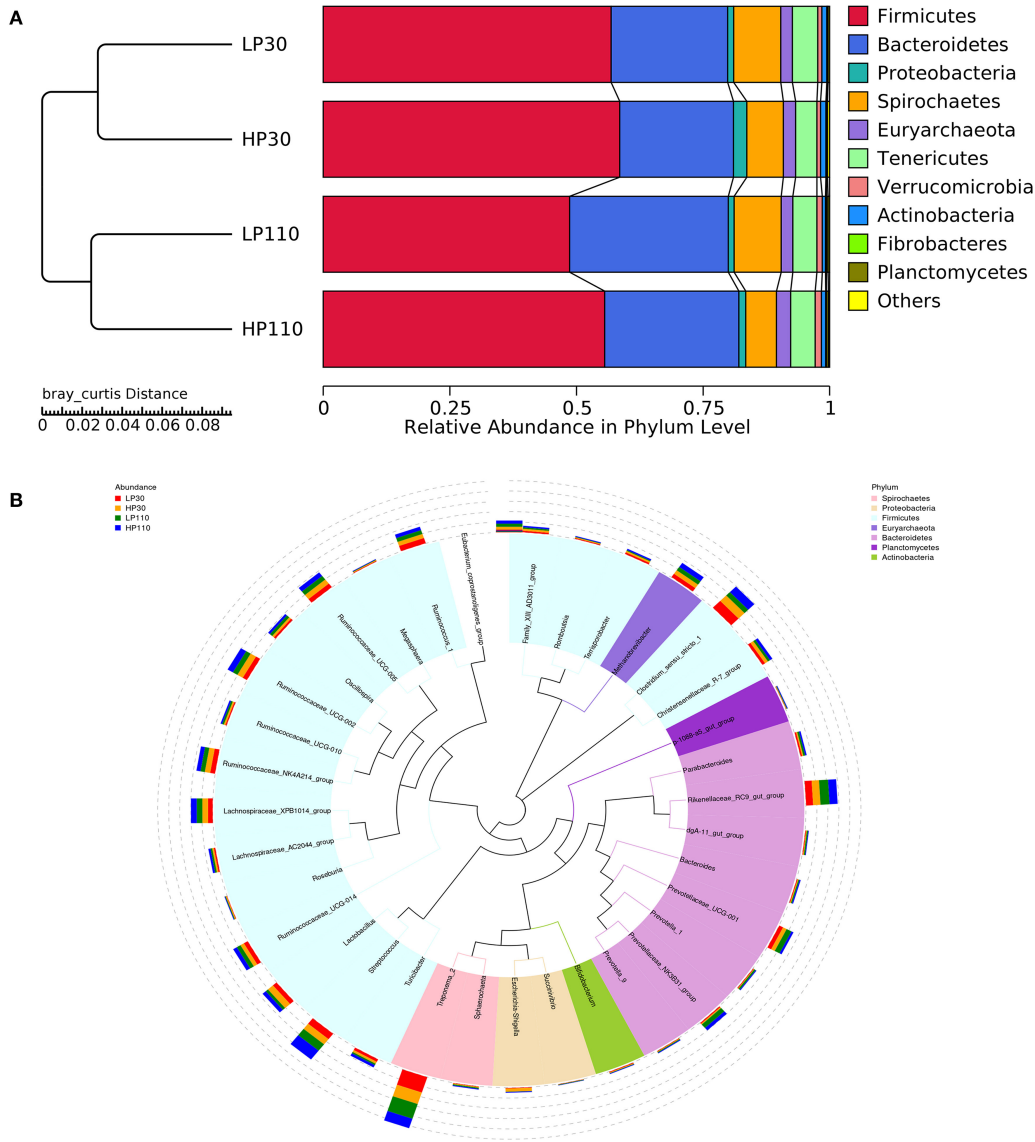
Changes of the relative abundance at phylum and genus levels. **(A)** Unweighted pair-group method with arithmetic mean (UPGMA) clustering analysis with the Bray-Curtis distance. The left panel shows the phylogenic tree, and the right panel displays the relative abundance of each group at the phylum level. **(B)** The phylogenetic tree constructed based on the sequence of the top 35 genera. The branches with different colors in the inner circle represent their corresponding phylum, and the stacked column chart in the outer circle indicates the relative abundance of each genus in different treatments. LP30 and LP110: sows with low-reproductive performance on d 30 and 110 of gestation, respectively; HP30 and HP110: sows with high-reproductive performance on d 30 and 110 of gestation, respectively. *n* = 13 for each group.

**Figure 5:**
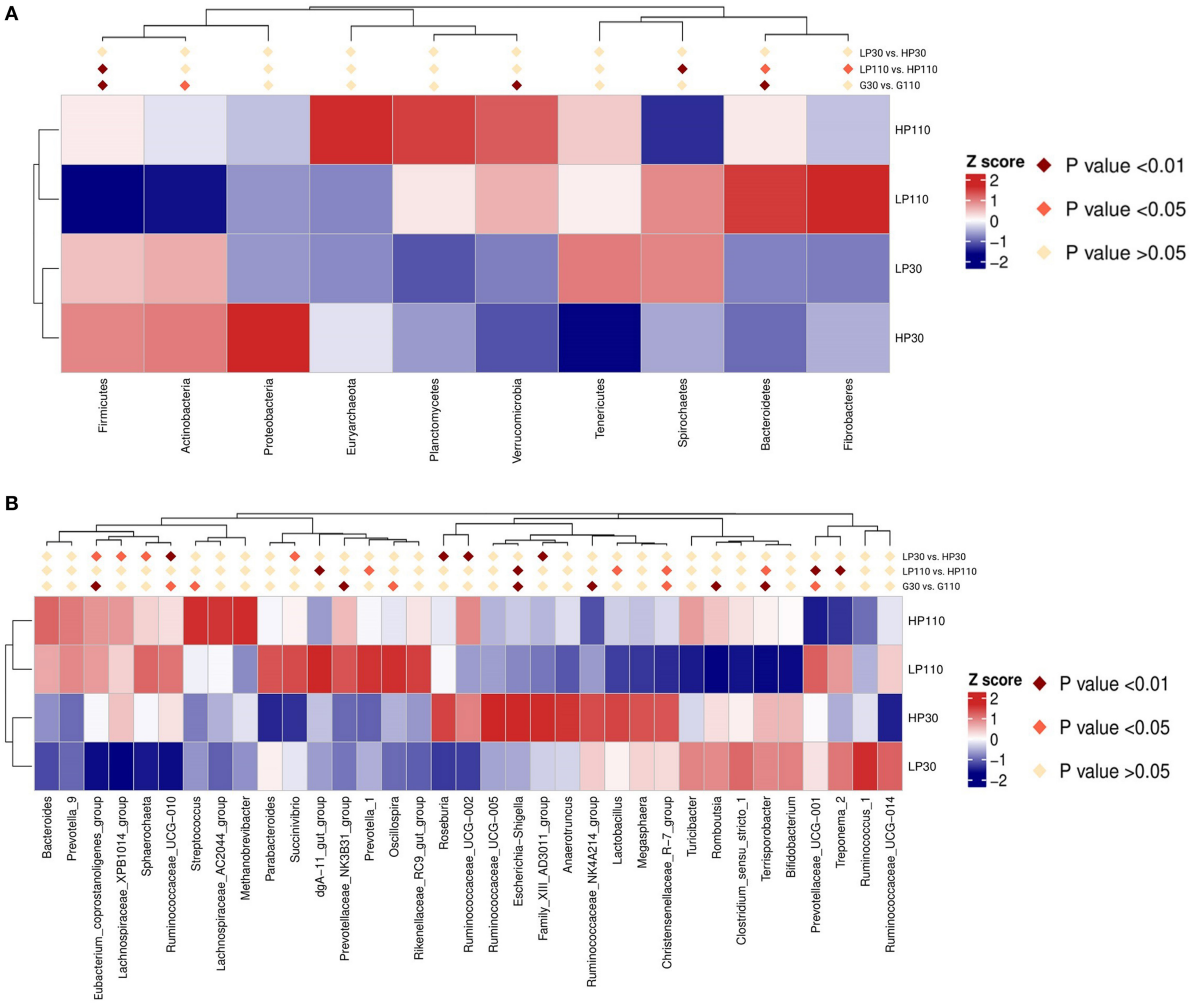
Heatmap distribution of OTUs in sow feces for all groups. **(A)** Comparison of the relative abundances of sow fecal microbiota in the top 10 at the phylum level. **(B)** Comparison of the relative abundances of sow fecal microbiota in the top 35 at the genus level. Different colors show the relative abundance of taxa. Fecal bacterial abundances were standardized with Z-score prior to the analyses. Positive z-scores reflect abundances above the average, whereas a negative z-score indicates abundance below the average. LP30 and LP110: sows with low-reproductive performance on d 30 and d 110 of gestation, respectively; HP30 and HP110: sows with high-reproductive performance on d 30 and d 110 of gestation, respectively. *n* = 13 for each group.

As shown in Figure 4, Firmicutes and Bacteroidetes were the most predominant phyla which accounted for more than 75%, followed by Spirochaetes and Tenericutes, in both groups during gestation (Figure 4A). No significant differences were observed in the top 10 phyla which accounted for more than 99.5% of the total bacteria population between the LP and HP groups on d 30 of gestation (*P* > 0.05, Figure 5A). However, the relative abundance of Firmicutes in the HP groups was significantly higher (*P* < 0.05) than that in the LP group, while the relative abundances of Bacteroidetes, Spirochaetes, and Fibrobacteres were significantly lower (*P* < 0.05) than that in the LP group on d 110 of gestation. In addition, the relative abundances of Firmicutes and Actinobacteria were significantly decreased (*P* < 0.05), while Bacteroidetes and Verrucomicrobia were significantly increased from gestation d 30 to d 110 (*P* < 0.05).

The species phylogenetic tree evolution was constructed by multiple sequences alignments to obtain the representative sequence of the top 35 genera. As shown in Figure 4B, the relative abundance of Firmicutes was contributed by *Clostridium_sensu_stricto_1, Streptococcus, Lactobacillus, Christensenellaceae_R-7_group, Ruminococcaceae_UCG-002, Ruminococcaceae_NK4A214_group, Ruminococcaceae_UCG-005, Ruminococcaceae_UCG-014, Ruminococcus_1*, and *Lachnospiracea*e_XPB1014_group; Bacteroidetes mainly distributed with *Rikenellaceae_RC9_gut_group, Prevotellaceae_UCG-001*, and *Prevotellaceae_NK3B31_group*; Spirochaetes was dominated by *Treponema_2*. In the LP and HP groups, *Treponema_2* and *Clostridium_sensu_stricto_1* were the top two genera on d 30 of gestation, and *Treponema_2* and *Streptococcus* were the most dominant on d 110 of gestation. Of the top 35 genera, compared with sows in the LP group, sows in the HP group had significantly higher (*P* < 0.05) relative abundances of *Eubacterium_coprostanoligenes_group, Lachnospiraceae_XPB1014_group, Sphaerochaeta, Ruminococcaceae_UCG-010, Roseburia, Ruminococcaceae_UCG-002*, and *Family_XIII_AD3011_group*, and significantly lower (*P* < 0.05) *Succinvibrio* on d 30 of gestation; sows in the HP group had significantly lower (*P* < 0.05) *Treponema_2, Prevotellaceae_UCG-001, Prevotella_1* and *dgA-11_gut_group*, as well as significantly higher (*P* < 0.05) *Lactobacillus, Christensenellaceae_R-7_group, Terrisporobacter*, and *Escherichia*-*Shigella* on d 110 of gestation (Figure 5B). Besides, sow fecal samples from gestation d 110 had significantly higher abundances of *Streptococcus, Prevotellaceae_NNK3B31_group, Oscillospira, Eubacterium_coprostanoligenes_group*, and *Ruminococcaceae_UCG-010*, but significantly lower *Prevotellaceae_UCG-001, Terrisporobacter, Escherichia*-*Shigella, Ruminococcaceae_NK4A214_group, Christensenellaceae_R-7_group*, and *Romboutsia* than those of sow fecal samples from gestation d 30 (*P* < 0.05).

### Correlation Analysis Between Sow Reproductive Performance and Fecal Microbiota

On day 30 of gestation, at the phylum level, the relative abundances of Firmicutes and Actinobacteria tended to be positively correlated with litter size (*P* < 0.10), while the relative abundance of Proteobacteria tended to be negatively correlated with litter size (*P* < 0.10); at the genus level, the relative abundances of *Turicibacter* and *Ruminococcaceae_UCG-014* had significant positive correlations with litter size (*P* < 0.05), and the relative abundances of *Clostridium_sensu_stricto_1* and *Romboutsia* tended to be positively correlated with litter size (*P* < 0.10, Table 2).

**Table 2:** The Spearman's correlation test between the sow fecal microbiota and litter size.

**Phase of gestation**	**Phylum**	**Genus**
D 30 of gestation	Firmicutes (0.351^+^)	*Clostridium_sensu_stricto_1* (0.376^+^)
		*Turicibacter* (0.479^*^)
		*Ruminococcaceae_UCG-014* (0.533^**^)
		*Romboutsia* (0.346^+^)
	Proteobacteria (−0.331^+^)	-
	Actinobacteria (0.369^+^)	-
D110 of gestation	Firmicutes (0.492^*^)	*Lactobacillus* (0.365^+^)
		*Clostridium_sensu_stricto_1* (0.434^*^)
		*Turicibacter* (0.445^*^)
		*Terrisporobacter* (0.466^*^)
		*Christensenellaceae_R-7_group* (0.406^*^)
	Bacteroidetes (−0.402^*^)	*Rikenellacea*e_RC9_gut_group (−0.495^*^)
	Spirochaetes (−0.526^**^)	*Treponema_2* (−0.490^*^)
		*Sphaerochaeta* (−0.347^+^)
	Proteobacteria (0.365^+^)	*Escherichia*-*Shigella* (0.578^**^)
	Actinobacteria (−0.627^**^)	-

+ *The correlation tends to be significant at a level of 0.10;*

* *the correlation is significant at a level of 0.05;*

** *the correlation is significant at a level of 0.01*.

On d 110 of gestation, at the phylum level, significant positive correlation between the relative abundance of Firmicutes and litter size was observed (*P* < 0.05), and the relative abundances of Bacteroidetes, Spirochaetes, and Actinobacteria were all significantly negatively correlated with litter size (*P* < 0.05); at the genus level, *Clostridium_sensu_stricto_1, Turicibacter, Terrisporobacter, Christensenellaceae_R-7_group*, and *Escherchia*-*Shigella* exhibited the significantly positive correlations with litter size (*P* < 0.05), while *Rikenellaceae_RC9_gut_group, Treponema_2*, and *Sphaerochaeta* had significant negative correlations with litter size (*P* < 0.05). In addition, the phylum Proteobacteria and genus *Lactobacillus* displayed a tendency to be positively correlated with litter size (*P* < 0.10), and the genus *Sphaerochaeta* tended to be negatively correlated with litter size (*P* < 0.10).

### Changes of Fecal SCFAs Concentrations During Gestation

**Figure 6:**
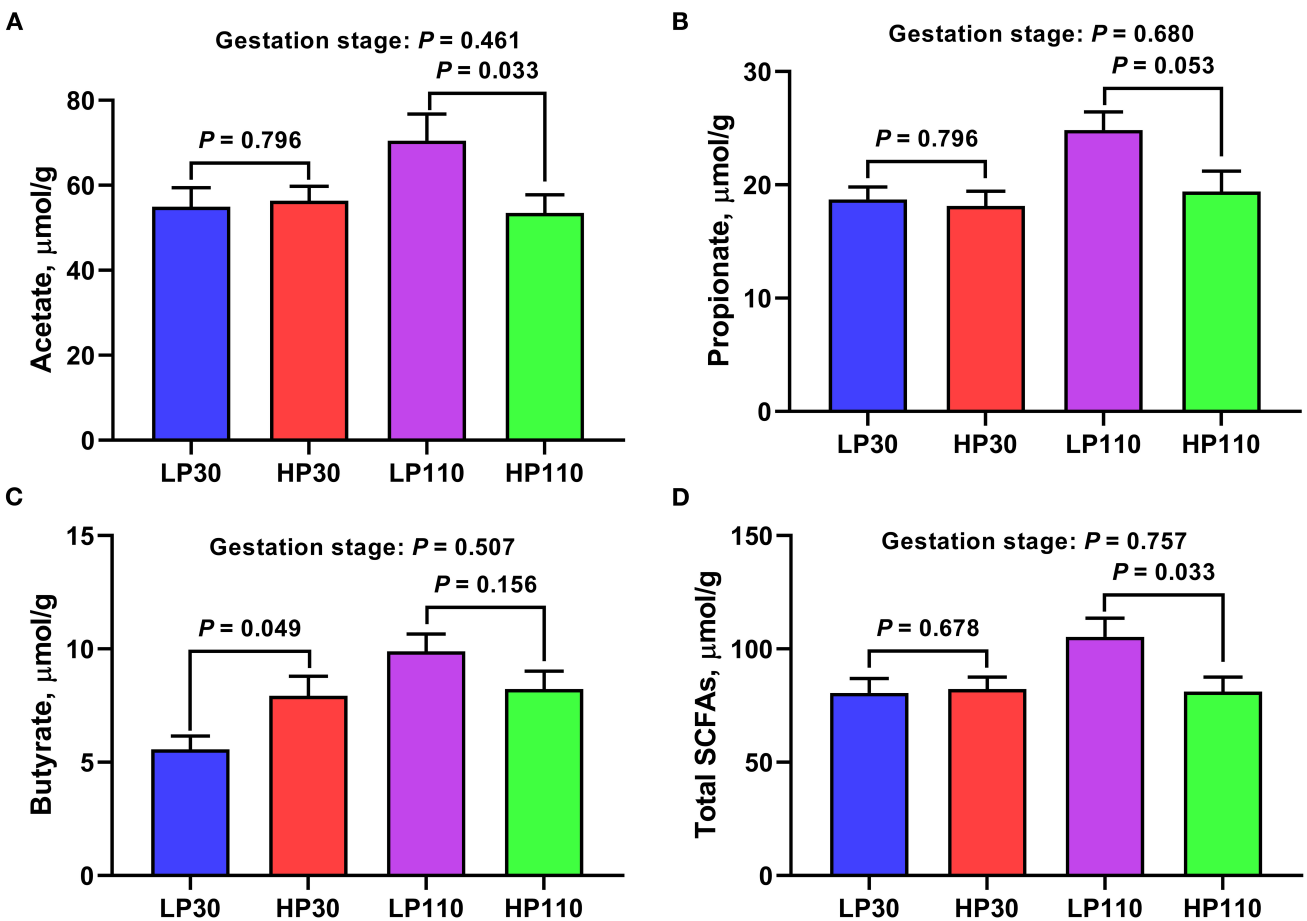
Fecal short-chain fatty acids (SCFAs) concentrations in low- and high-reproductive performance groups on d 30 and 110 of gestation. **(A)** Acetate. **(B)** Propionate. **(C)** Butyrate. **(D)** Total SCFAs. Total SCFAs, the sum of acetate, propionate, and butyrate. LP30 and LP110: sows with low-reproductive performance on d 30 and 110 of gestation, respectively; HP30 and HP110: sows with high-reproductive performance on d 30 and 110 of gestation, respectively. Gestation stage: difference in the variations between gestation d 30 and d 110. Values are mean ± standard error (*n* = 13).

The concentrations of fecal short-chain fatty acids on d 30 and d 110 of gestation in the two groups are listed in Figure 6. On d 30 of gestation, there were no significant differences in fecal acetate, propionate, and total SCFAs concentrations between the LP group and HP group (*P* > 0.05), but sows from HP group showed significantly higher butyrate concentration than those of sows from LP group (*P* < 0.05). On d 110 of gestation, sows in the HP group had significantly lower acetate and total SCFAs concentrations than sows in the LP group (*P* < 0.05), and the propionate concentration in the HP group tended to be lower than that in the LP group (*P* = 0.053). The fecal SCFAs concentrations of sows on d 110 of gestation did not differ with that of sows on d 30 of gestation (*P* > 0.05).

### Changes in Plasma Metabolites During Gestation

**Figure 7:**
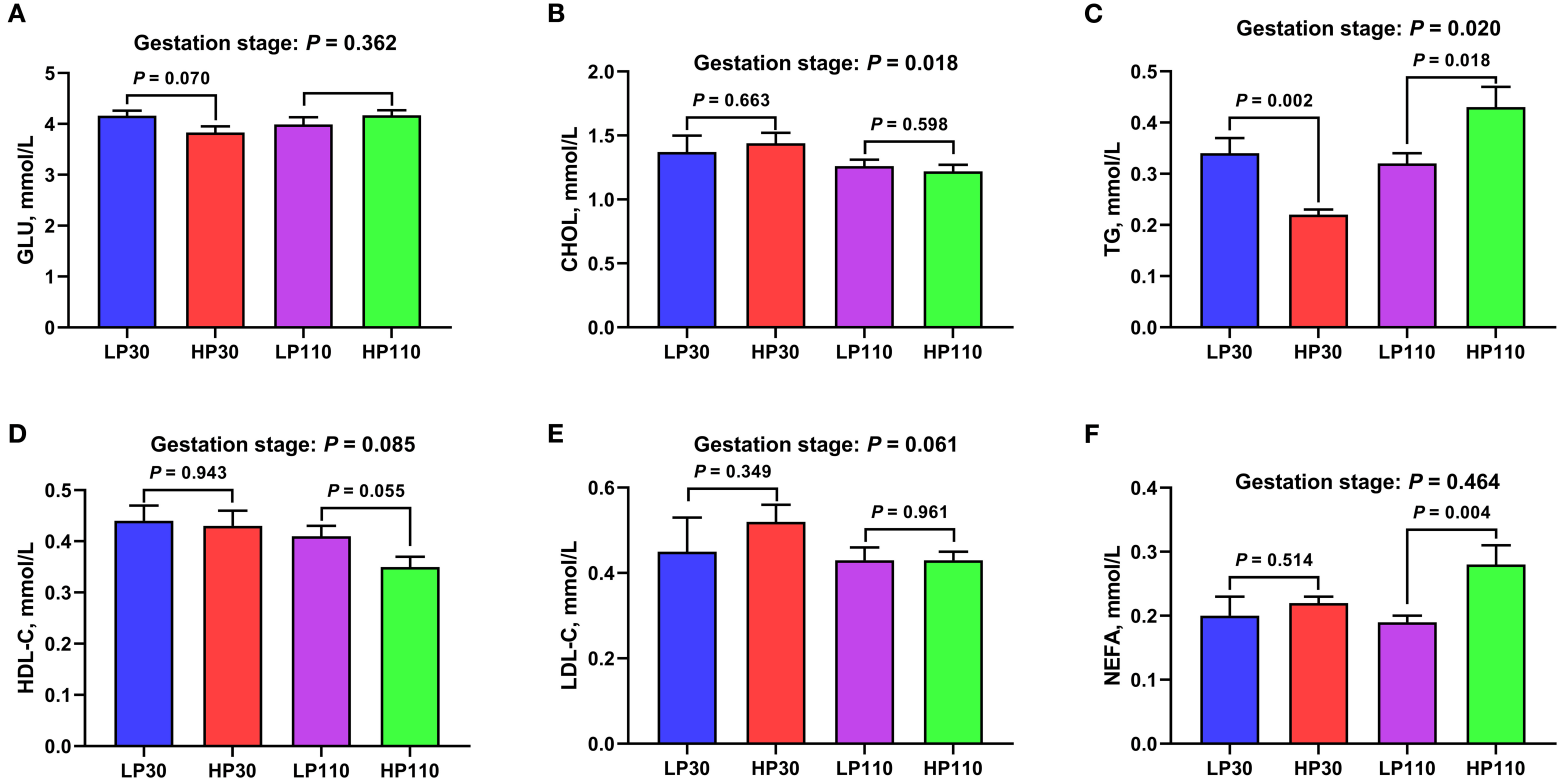
The levels of plasma metabolites in low- and high-reproductive performance groups on d 30 and 110 of gestation. **(A)** Glucose (GLU); **(B)** Cholesterol (CHOL); **(C)** Triglyceride (TG); **(D)** High density lipoprotein cholesterol (HDL-C); **(E)** Low density lipoprotein cholesterol (LDL-C); **(F)** Non-esterified fatty acid (NEFA). LP30 and LP110: sows with low-reproductive performance on d 30 and 110 of gestation, respectively; HP30 and HP110: sows with high-reproductive performance on d 30 and 110 of gestation, respectively. Gestation stage: difference in the variations between gestation d 30 and d 110. Values are mean ± standard error (*n* = 13).

As shown in Figure 7, on day 30 of gestation, significantly lower plasma TG levels were observed in the HP group compared with those in the LP group (*P* < 0.05); sows in the HP group tended to have a lower plasma GLU concentration than sows in the LP group (*P* = 0.070). On d 110 of gestation, sows in the HP group had significantly higher plasma TG and NEFA levels (*P* < 0.05) and tended to have lower plasma HDL-C concentration compared with those of sows in the LP group (*P* = 0.055). Besides, the concentrations of CHOL (*P* = 0.018), HDL-C (*P* = 0.085), and LDL-C (*P* = 0.061) were decreased, and the levels of TG (*P* = 0.020) were increased from gestation d 30 to d 110.

### Changes of Plasma Inflammatory Factors and Immunoglobulins During Gestation

**Figure 8:**
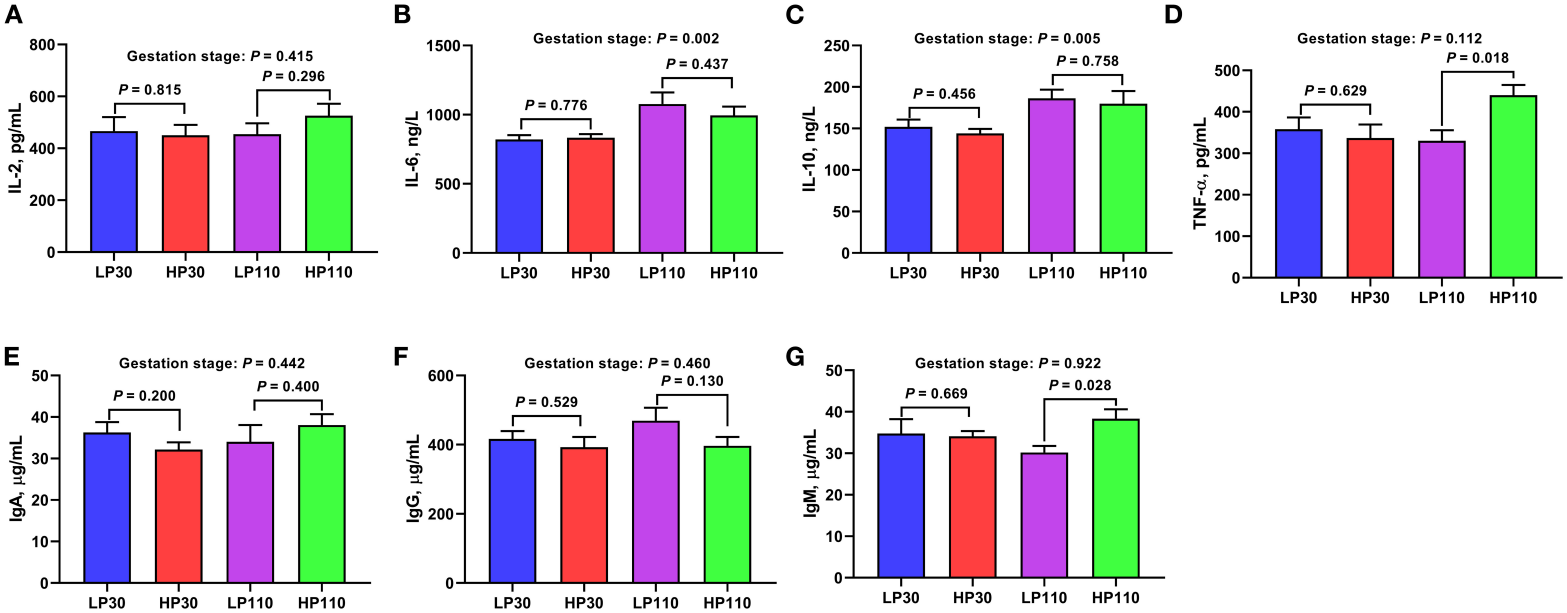
The levels of inflammatory factors and immunoglobulins in low- and high-reproductive performance groups on d 30 and 110 of gestation. **(A)** Interleukin-2 (IL-2); **(B)** Interleukin-6 (IL-6); **(C)** Interleukin-10 (IL-10); **(D)** Tumor necrosis factor-α (TNF-α); **(E)** Immunoglobulin A (IgA); **(F)** Immunoglobulin G (IgG); **(G)** Immunoglobulin M (IgM). LP30 and LP110: sows with low-reproductive performance on d 30 and 110 of gestation, respectively; HP30 and HP110: sows with high-reproductive performance on d 30 and 110 of gestation, respectively. Gestation stage: difference in the variations between gestation d 30 and d 110. Values are mean ± standard error (*n* = 13).

The levels of plasma inflammatory factors and immunoglobulins are shown in Figure 8. There were no significant differences in the plasma concentrations of inflammatory factors and immunoglobulins on d 30 of gestation between the LP and HP groups (*P* > 0.05). However, significantly higher TNF-α and IgM concentrations were observed in the HP group compared with those in the LP group on d 110 of gestation (*P* < 0.05). In addition, the plasma IL-6 and IL-10 levels were significantly increased (*P* < 0.05) from gestation d 30 to d 110.

### Changes of Plasma Hormone Contents During Gestation

**Figure 9:**
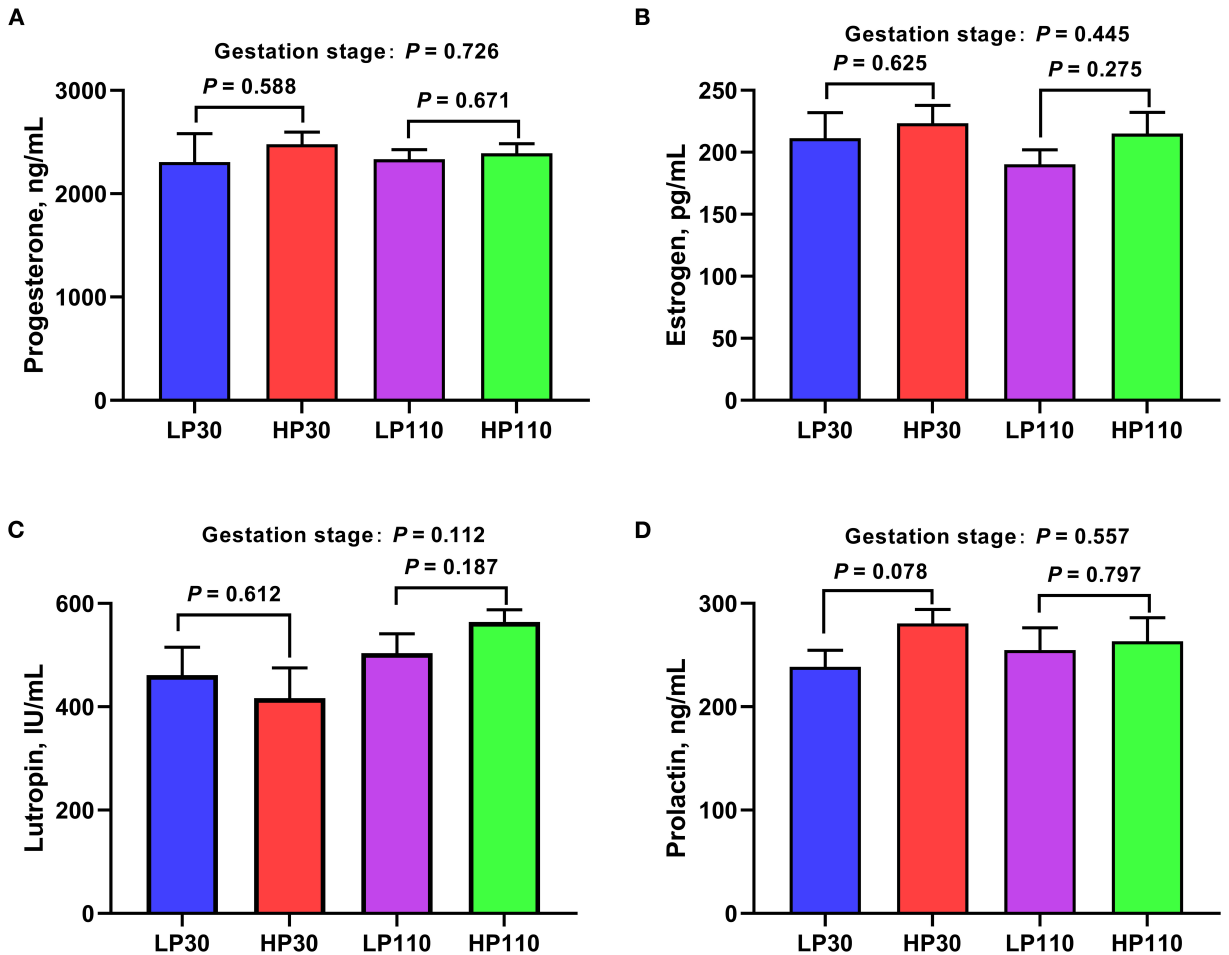
The plasma concentrations of reproductive hormones in low- and high-reproductive performance groups on d 30 and 110 of gestation. **(A)** Progesterone; **(B)** Estrogen; **(C)** Lutropin; **(D)** Prolactin. LP30 and LP110: sows with low-reproductive performance on d 30 and 110 of gestation, respectively; HP30 and HP110: sows with high-reproductive performance on d 30 and 110 of gestation, respectively. Gestation stage: difference in the variations between gestation d 30 and d 110. Values are mean ± standard error (*n* = 13).

The plasma hormone contents are shown in Figure 9. There were no significant differences in the plasma hormone concentrations on d 30 and d 110 of gestation between the LP and HP groups (*P* > 0.05). The plasma hormone contents of sows on gestation d 110 did not differ from those of sows on gestation d 30 (*P* > 0.05).

### Correlation Analysis Between Fecal Microbial Abundance and Plasma Biochemical Indices

**Figure 10:**
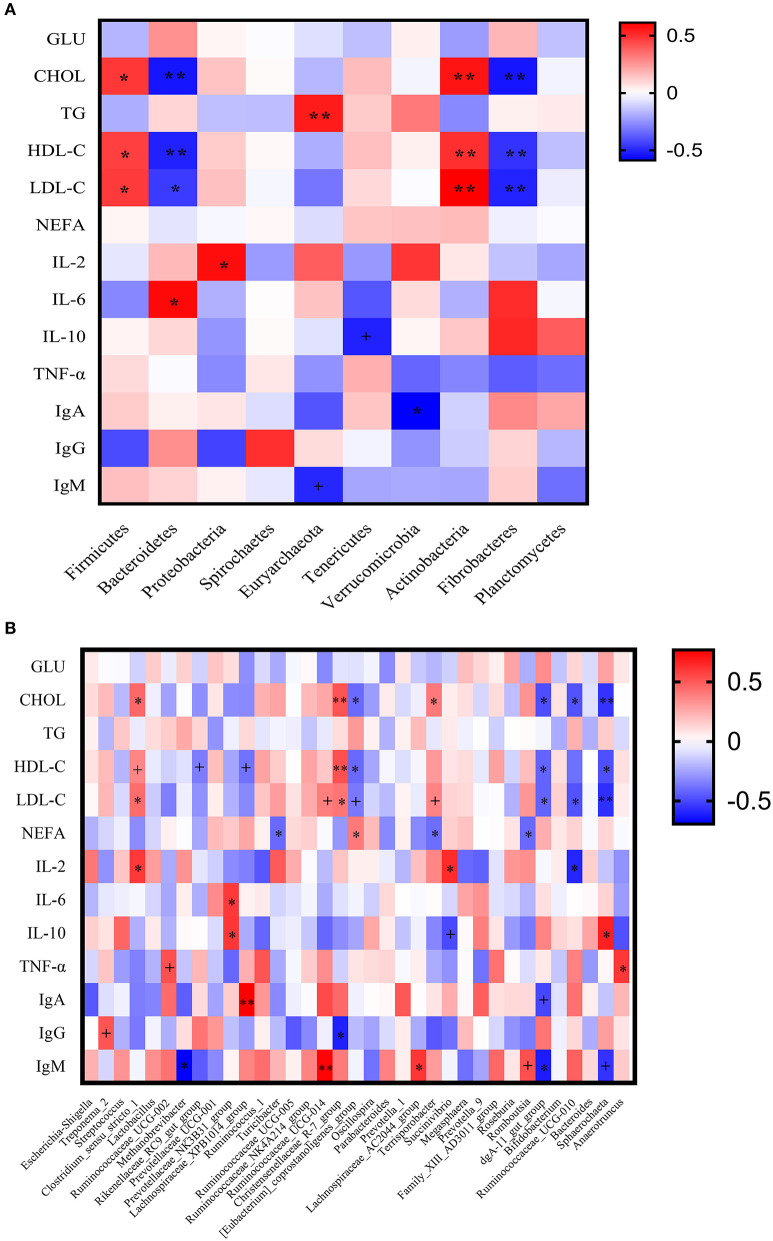
Correlation analysis between the plasma biochemical indices and sow fecal microbiota. **(A)** At phylum level; **(B)** At genus level. GLU, Glucose; CHOL, cholesterol; TG, triglyceride; HDL-C, high density lipoprotein cholesterol, LDL-C, low density lipoprotein cholesterol; NEFA, non-esterified fatty acid; IL-2, interleukin-2; IL-6, interleukin-6; IL-10, interleukin-10; TNF-α, tumor necrosis factor-α; IgA, immunoglobulin A; IgG, immunoglobulin G; IgM, immunoglobulin M. ^+^The correlation tends to be significant at a level of 0.10; *the correlation is significant at a level of 0.05; ** the correlation is significant at a level of 0.01.

At the phylum level (Figure 10A), the plasma levels of CHOL, HDL-C, and LDL-C showed positive correlations with the abundances of Firmicutes and Actinobacteria (*P* < 0.05) and negative correlations with the abundances of Bacteroidetes and Fibrobacteres (*P* < 0.05); the plasma TG level had significant positive correlation with Euryarchaeota abundance (*P* < 0.05); the plasma IL-2 concentration was significantly positively correlated with Proteobacteria abundance (*P* < 0.05); the plasma IL-6 concentration was significantly positively correlated with the abundance of Bacteroidetes (*P* < 0.05); the plasma IgA concentration had significant negative correlation with Verrucomicrobia (*P* < 0.05). In addition, the IL-10 concentration tended to be negatively correlated with the abundance of Tenericutes (*P* < 0.10), and the IgM concentration tended to be negatively correlated with the abundance of Euryarchaeota (*P* < 0.10).

At the genus level (Figure 10B), the plasma level of CHOL showed positive correlations with the abundances of *Clostridium_sensu_stricto_1, Christensenellaceae_R-7_group*, and *Terrisporobacter* (*P* < 0.05) and negative correlations with the abundances of *Eubacterium_coprostanoligenes_group, dgA-11_gut_group, Ruminococcaceae_UCG-010*, and *Sphaerochaeta* (*P* < 0.05). The plasma HDL-C concentration was significantly positively correlated with the abundance of *Christensenellaceae_R-7_group* (*P* < 0.05) and was significantly negatively correlated with the abundances of *Eubacterium_coprostanoligenes_group, dgA-11_gut_group*, and *Sphaerochaeta* (*P* < 0.05). The plasma LDL-C level had significant positive correlations with the abundances of *Clostridium_sensu_stricto_1* and *Christensenellaceae_R-7_group* (*P* < 0.05) and had significant negative correlations with the abundances of *dgA-11_gut_group, Ruminococcaceae_UCG-010*, and *Sphaerochaeta* (*P* < 0.05). The plasma NEFA concentration displayed positive correlation with the abundance of *Eubacterium_coprostanoligenes_group* and negative correlations with the abundances of *Turicibacter, Terrisporobacter*, and *Romboutsia* (*P* < 0.05). The plasma IL-2 concentration was significantly positively correlated with the abundances of *Clostridium_sensu_stricto_1* and *Succinivibrio*, and significantly negatively correlated with the abundance of *Ruminococcaceae_UCG-010* (*P* < 0.05). The plasma IL-6 level showed positive correlation with the *Prevotellaceae_NNK3B31_group* abundance (*P* < 0.05). The plasma IL-10 concentration had significant positive correlations with the abundances of *Prevotellaceae_NNK3B31_group* and *Sphaerochaeta* (*P* < 0.05). The plasma TNF-α concentration was significantly positively correlated with the abundances of *Anaerotruncus* (*P* < 0.05). The plasma level of IgA showed positive correlation with the *Lachnospiraceae_XPB1014_group* abundance (*P* < 0.05). The plasma level of IgG indicated negative correlation with the *Christensenellaceae_R-7_group* abundance (*P* < 0.05). The plasma IgM concentration was significantly positively correlated with the abundances of *Ruminococcaceae_UCG-014* and *Lachnospiraceae_AC2044_group* (*P* < 0.05), and was significantly negatively correlated with the abundances of *Methanobrevibacter* and *dgA-11_gut_group* (*P* < 0.05).

## Discussion

Maternal metabolism changes dramatically during the gestation period. Especially, maternal glucose and lipid metabolism plays a vital role in the initiation and development of gestation (26). The early stage of gestation can be regarded as an anabolic state to meet the fetal-placental and maternal demands of late gestation and lactation, with an increase in maternal fat stores and small increases in insulin sensitivity (27). The present study showed that the sows in the HP group had lower plasma levels of GLU and TG than those in LP group on d 30 of gestation. Plasma levels of GLU and TG are important indicators of glycolipid metabolism. Plasma lipid profiles at early pregnancy may predict the incidence and severity of pre-eclampsia in humans (28). The previous study in humans showed that higher plasma GLU concentration in the first trimester of pregnancy was a risk factor for adverse perinatal and neonatal outcomes, such as diabetes-related complications, gestational hypertension, and obesity (29). Similarly, a study in dairy cows demonstrated that high glucose levels at early gestation had an adverse impact on early embryonic development (30). The reason might be related to high nutritional level that increased the metabolic clearance rate of progesterone (31). The results might suggest that higher glucose level was not conducive to the development of embryos. Besides, higher plasma TG concentration is usually associated with abnormal lipid metabolism and causally related to an increased risk of cardiovascular disease in the clinic (32). Previous research indicated that higher plasma TG concentration demonstrated a poor health status of a gestating sow (33). Therefore, the sows in HP group is in a better physical state than those in LP group on d 30 of gestation.

In contrast, the sows in HP group showed higher plasma levels of NEFA and TG on d 110 of gestation. Late pregnancy is characterized as a catabolic state with increased insulin resistance which leads to increases in concentrations of maternal glucose and NEFA in plasma, allowing for greater substrate availability for rapid fetal development (27, 34). Serum NEFA, one of the most important biomarkers of energy balance status, is the product of lipolysis of storage fat, such as TG. Elevated plasma NEFA level mediates many adverse metabolic effects, including obesity, insulin resistance, hypertension, and chronic inflammation (35–38). Consistently, increased plasma TNF-α concentration was found in the sows of HP group on d 110 of gestation. Tumor necrosis factor-alpha is a highly pleiotropic cytokine and is thought of as a vital mediator of inflammatory responses, metabolic activation, and cell death (39). The results of the present study demonstrated that HP sows might be in a more dramatic catabolic status to ensure the normal growth and development of the fetus during late gestation, leading to greater inflammation than LP sows, which was in accord with previous results in Shao et al. (40).

It is well-known that the dramatic changes of the microbial community can usually affect the health status of the host. In the present study, higher observed species, Shannon index, and Chao 1 index, as well as OTUs number, which was used to assess fecal microbial community richness and diversity, were observed in the HP group compared with the LP group on d 30 of gestation. Gut microbial diversity has been regarded as a new biomarker of health and metabolic capacity and low microbial diversity was often associated with poor health status such as inflammatory response, oxidative stress, and obesity (41, 42). In addition, sows in the HP group had the higher abundances of *Eubacterium_coprostanoligenes_group, Lachnospiraceae_XPB1014_group, Ruminococcaceae_UCG-010, Roseburia*, and *Ruminococcaceae_UCG-002* on d 30 of gestation. *Eubacterium coprostanoligenes* is a cholesterol-reducing bacterium and inversely correlated with the inflammatory response (43, 44). Li et al. (45) found that feeding *Eubacterium coprostanoligenes* to germ-free mice decreased blood CHOL concentration. Consistently, the correlation analysis in the present study also demonstrated that the relative abundance of *Eubacterium_coprostanoligenes_group* was negatively correlated with plasma CHOL concentration. Lachnospiraceae family are abundant in healthy humans (46) and can impact their hosts by producing SCFAs, converting primary to secondary bile acids, and competitively inhibiting colonization of intestinal pathogens (47, 48). *Ruminococcaceae*, which has carbohydrate-active enzymes, sugar transport mechanisms, and metabolic pathways for the degradation of complex plant materials (49), is a common digestive tract microbe. Fomenky et al. (50) showed that *Ruminococcaceae* might enhance mucus production and benefit to improve inflammatory responses in calves. In the present study, *Ruminococcaceae_UCG-010* was shown to be negatively associated with the plasma concentration of proinflammatory factor IL-2. *Roseburia* is a prominent gut-associated butyrate-producing genus (51) and inversely correlated with many diseases, such as inflammatory bowel disease (52) and atherosclerotic lesion (53). Consistently, increased fecal butyrate concentration was found in sows in the HP group. Microbial-driven butyrate has been shown to exhibit protective effects toward inflammatory diseases (54). Previous study has shown that butyrate oxidation can make up around 70 and 60% of the oxygen consumption in human descending colon and ascending colon, and inhibit the proliferation of aerobic pathogens (55). These findings might partly explain the better health status of HP sows at early gestation.

Interestingly, the significant difference in alpha diversity disappeared, but significant difference was observed in beta diversity between HP and LP groups on d 110 of gestation. This was in keeping with the results in Uryu et al. (12) who explored the relationship between sow productive capacities and the fecal microbiota in different farms. However, Shao et al. (40) reported that alpha diversity and beta diversity both differed between sows with high- and low-reproductive performance during late gestation. It might suggest that the beta diversity, not alpha diversity, was a critical factor to evaluate the effect of gut microbiota on sow reproductive performance (12).

In addition, compared with sows in the LP group, sows in the HP group had the lower abundances of Bacteroidetes (including *Prevotellaceae_UCG-001, Prevotella_1*, and *dgA-11_gut_group*) and Spirochaetes (*Treponema_2*) which were negatively correlated with litter size, but the higher abundance of Firmicutes (containing *Lactobacillus, Christensenellaceae_R-7_group*, and *Terrisporobacter*) and genus *Escherichia*-*Shigella* exhibited positive correlations with litter size on day 110 of gestation. In the present study, Firmicutes and Bacteroidetes were the most predominant phyla, regardless of the stage of gestation, which were in accordance with previous studies on sows (40, 56, 57). A previous study in obese children showed that the abundance of Firmicutes had the positive association with plasma TNF-α level (58). *Bacteroidetes*, as well as *Treponema_2*, includes a large number of cellulases, glycoside hydrolases, glycosyl transferases, and have the capacity to degrade polymers such as cellulose, hemicellulose, and lignin (59, 60), which might be the reason for the decreases in fecal concentrations of acetate, propionate, and total SCFAs. Previous studies indicated that a changed gut microbiota characterized by increased levels of Firmicutes and depleted Bacteroidetes was associated with chronic or low-grade inflammation (11). *Escherichia*-*Shigella*, belonging to phylum *Proteobacteria*, is generally taken as non-pathogenic bacteria and can become pathogenic bacteria when stimulated by stress (61). Shao et al. (40) also reported that predicted metabolic functions related to lipopolysaccharide biosynthesis significantly higher in HP sows than in LP sows during late gestation. The greater production of total SCFAs and propionate on d 110 of gestation in the LP group may be a compensatory mechanism in order to ensure the survival of fetuses and try to reduce pathogenic microorganisms, which need to be further studied. Moreover, Koren et al. (10) showed that dramatical alterations of species and abundance of gut microbiota contributed to the metabolic changes during gestation which was characterized by greater adiposity and insulin resistance to meet the needs of the rapid growth of fetuses during late gestation in human. Therefore, it might suggest that the alteration in gut microbiota during late gestation, associated with the increases in plasma TG and NEFA, in sows with high-reproductive performance might be more conducive to the growth and development of the fetus.

Interestingly, we also found increased abundance of *Terrisporobacter* that had significant negative correlations with the plasma NEFA concentration, which might be helpful to decrease the plasma NEFA from the HP sow and resist inflammatory response during late gestation. We also found increased plasma IgM concentration in HP sows on d 110 of gestation, which might be related to the increased abundance of *Lactobacillus* and the decreased abundance of *dgA-11_gut_group*. Wang et al. (62) reported that *Lactobacillus* supplementation in weanling piglets could increase plasma level of IgM. Immunoglobulin M, serving as the first line of host defense against infections, is the first antibody isotype to appear during immune responses and plays a vital role in immune regulation and immunological tolerance (63). The abundance of *dgA-11_gut_group* was negatively correlated with the plasma IgM concentration in the present study. This might be an important reason that the microecological balance of the intestinal tract of HP sows could restore during lactation (40).

In addition, we explored the shifts in plasma parameters, fecal metabolites, and microbiota from gestation d 30 to d 110 in the present study. The results indicated that plasma level of TG was increased, but levels of CHOL, HDL-C, and LDL-C were reduced on d 110 of gestation. Ji et al. (56) also showed that plasma concentrations of total CHOL and HDL-C were reduced from gestation d 60 to d 110. It suggested that lipid metabolism in the hepatic and adipose tissues of sows were activated to maintain the nutritional needs of the fetus in late gestation. Dramatic switches in lipid catabolism were often associated with inflammatory responses (64). Consistently, plasma concentrations of IL-6 and IL-10 were both elevated on d 110 of pregnancy. Interleukin-6 is a pleiotropic pro-inflammatory cytokine and involved in chronic inflammation and immune regulatory cascades (65). Interleukin-10, a prototypical anti-inflammatory cytokine produced by CD4 (+) cells, plays an important role in inhibiting inflammatory reaction by suppressing the upstream activities of antigen presenting cells and T cell functions (66). Increased serum concentration of IL-6 frequently accompanied an increased level of IL-10 in serum under inflammatory conditions (67). The alteration of abundances of phyla Firmicutes, Bacteroidetes, and Verrucomicrobia was in keeping with the results in Zhou et al. (11) that Firmicutes was significantly decreased while Bacteroidetes and Verrucomicrobia increased from d 30 to d 110 of gestation. However, Zhou et al. (11) observed an increase in Actinobacteria at late gestation, which was in line with Liu et al. (68). It suggested that the changes of abundance of Actinobacteria might be not associated with the progress of gestation. In terms of the genus level, the relative abundances of fecal *Streptococcus, Oscillospira*, and *Ruminococcaceae_UCG-010* were increased, and that of fecal *Terrisporobacter* was decreased with progression of pregnancy. Zhou et al. (11) also showed increased *Oscillospira* and decreased *Terrisporobacter* in sow feces from gestation d 30 to d 110. The changes of abundances of *Terrisporobacter* and *Ruminococcaceae_UCG-010* were in accord with alteration of plasma CHOL concentration. *Streptococcus*, including Gram-positive organisms shaped in cocci and organized in chains, are commensals, pathogens, and opportunistic pathogens for humans and animals (69). Previous study in humans also reported that *Streptococcus* was enriched in late gestation compared to in early gestation (10). However, Zhou et al. (11) found a reduction in fecal *Streptococcus* from gestation d 30 to d 110. Therefore, further study is required to indentify which microbiome is involved in the progress of pregnancy. Interestingly, the SCFAs were not significantly altered during gestation although significant microbiota compositions occurred, which was consistent with Liu et al. (68) and Zhou et al. (11). Above all, the sows underwent dramatic metabolic changes over the course of a normal pregnancy, which was associated with the profound alteration of the gut microbiota.

## Conclusion

In summary, our findings demonstrated that microbial abundances and community structures differed significantly between sows with different litter sizes during gestation, which was associated with changes in plasma biochemical parameters, inflammatory factors, and immunoglobulin, as well as fecal metabolites. Besides, plasma biochemical parameters and cytokines shifted dramatically from gestation d 30 to d 110, which were associated with the alterations in microbial composition and diversity. These findings revealed that sow reproductive performance might be associated with the changes of maternal gut microbiota during gestation and provided a microbial perspective to improve sow reproductive performance in pig production.

## Data Availability Statement

The assembled HiSeq sequences obtained in the present study were submitted to National Center of Biotechnology Information (NCBI) Sequence Read Archive (SRA) under accession PRJNA721963 (Illumina sequences).

## Ethics Statement

The animal study was reviewed and approved by the Animal Care and Use Committee of Shandong Agricultural University.

## Author Contributions

YL: conceptualization, investigation, supervision, and writing—review and editing. JC: data curation, project administration, and writing—original draft. JC and FL: formal analysis. WY: funding acquisition. SJ and YL: methodology. JC and YL: software. FL, WY, and YL: validation. JC and SJ: visualization. All authors contributed to the article and approved the submitted version.

## Funding

This research was funded by the Starting Research Fund from the Shandong Agricultural University (040/72185) and the Shandong Province Pig Industry Technology System (SDAIT-08-04).

## Conflict of Interest

The authors declare that the research was conducted in the absence of any commercial or financial relationships that could be construed as a potential conflict of interest.

## Publisher's Note

All claims expressed in this article are solely those of the authors and do not necessarily represent those of their affiliated organizations, or those of the publisher, the editors and the reviewers. Any product that may be evaluated in this article, or claim that may be made by its manufacturer, is not guaranteed or endorsed by the publisher.
