# Supplementary material for: Comparison of Gut Microbiota and Metabolic Status of Sows With Different Litter Sizes During Pregnancy
Source: Front Vet Sci. 2021 Dec 23;8:793174. doi: 10.3389/fvets.2021.793174 (PMC8733392; doi:10.3389/fvets.2021.793174)
Supplement: Supplementary file 1 [file Data_Sheet_1.docx]

Supplementary Material

1. **Supplementary Data**

**SupplementaryTable1.** Ingredients and chemical composition of gestation diet.

| Items | Gestation diet |
| --- | --- |
| Ingredients, % |  |
| Corn | 65.99 |
| Soybean meal, 44% CP | 13.06 |
| Wheat bran | 18.00 |
| L-lysine HCl, 76.8% | 0.14 |
| L-threonine, 98% | 0.06 |
| Limestone | 0.99 |
| Monocalcium phosphate | 0.67 |
| Sodium chloride | 0.40 |
| Choline | 0.14 |
| Vitamin premix^1^ | 0.05 |
| Trace mineral premix^2^ | 0.5 |
| Total | 100 |
| Calculated analysis |  |
| Digestible energy, MJ/kg | 13.49 |
| Crude protein, % | 12.91 |
| Crude fat, % | 3.28 |
| Crude fiber, % | 3.05 |
| Ca, % | 0.61 |
| Available P, % | 0.27 |
| Lysine, % | 0.61 |
| Methionine, % | 0.18 |
| Threonine, % | 0.46 |
| Tryptophan, % | 0.13 |

^1^ Provided per kilogram of complete gestation diet: vitamin A 7500 IU, vitamin D_3_ 5000 IU, vitamin E 37.5 IU, vitamin K_3_ 5 mg, vitamin B_1_ 5 mg, vitamin B_2_ 12.5 mg, vitamin B_6_ 7.5 mg, vitamin B_12_ 0.05 mg, biotin 0.2 mg, niacin 50 mg, folic acid 2.5 mg and D-calcium pantothenate 25 mg.

^2^ Provided per kilogram of complete gestation diet: 100 mg of Fe as FeSO_4_, 100 mg of Zn as ZnSO_4_, 10 mg of Cu as CuSO_4_, 30 mg of Mn as MnSO_4_, 0.6 mg of I as KI and 0.25 mg of Se as Na_2_SeO_3_.


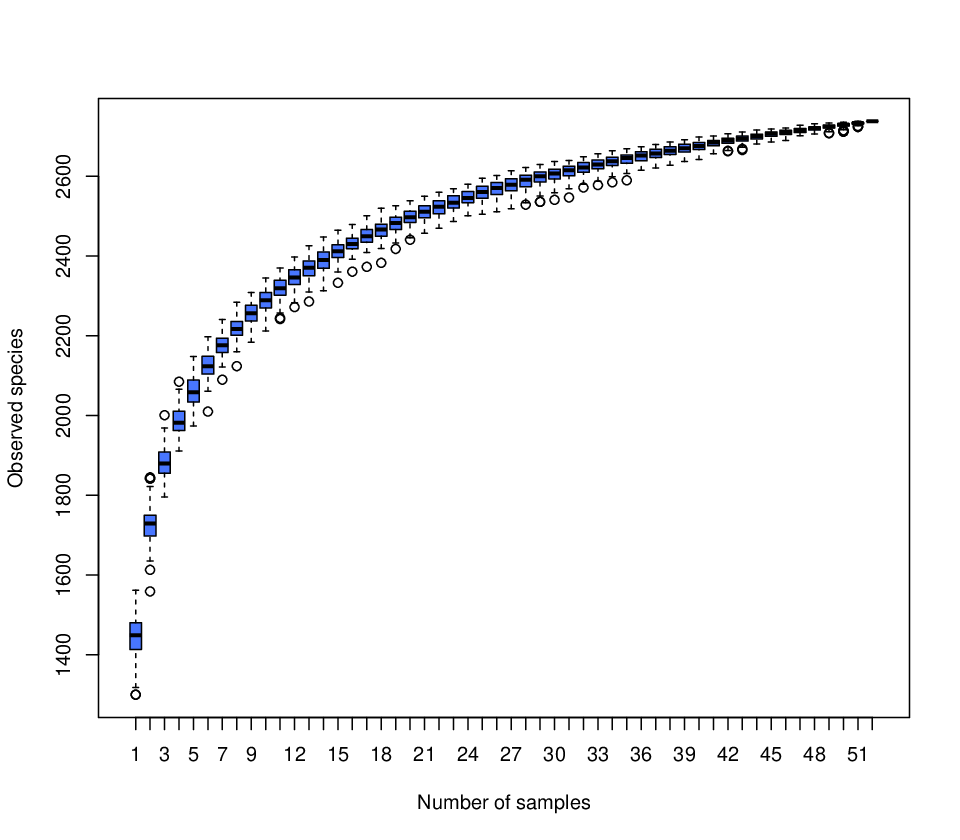


**SupplementaryFigure 1.**Species accumulation curves.

1. **Supplementary Methods**

**2.1 Analysis of Inﬂammatory Factors, Immunoglobulins, and Reproductive Hormones**

The prepared samples (50 μL) and diluted standard solutions were added to the corresponding microplates. After reacting for 30 min at 37 ℃, the microplates were washed five times and 50 μl of the HRP-Conjugated Reagent was added to each well. Then the microplates were cultured for 30 min at 37 ℃ and washed five times again. Chromogenic procedure was performed with two kinds of chromogenic agents for 10 min at 37 ℃ followed by Stop Buffer addition. Finally, the absorbance of each well was read within 15 min, and the concentrations of IL-2, IL-6, IL-10, TNF-α, IgA, IgG, IgM, , progesterone, estrogen, lutropin, and prolactin were calculated using the standard curve made with standard solutions.

**2.2 Determination of Fecal Short-chain Fatty Acids**

Fecal samples (about 0.7 g) were suspended in 1.5 mL of distilled water and allowed to stand for 30 min, followed by being centrifuged for 15 min at 15,000 × g at 4 ℃. Then 1 mL supernatant was transferred and mixed with metaphosphoric acid (0.2 mL, 25%, w/v) and crotonic acid (23.3 µL, 210 mmol/L). After standing at 4 ℃ for 30 min, the samples were centrifuged for 10 min at 15,000 × g again. Then the supernatant was transferred and mixed with chromatographic methanol (1:1, v/v). After centrifugation at 10,000 × g, an amount of 1 µL supernatant was analyzed using a gas chromatography (Varian CP-3800 GC, USA).
